# Spectroscopic Monitoring and Chemometric-Based Feedback Control of the Diazotization Reaction

**DOI:** 10.1021/acs.oprd.6c00030

**Published:** 2026-07-09

**Authors:** Máté Fent, Attila Farkas, Hajnalka Pataki, Zsombor K. Nagy, István Csontos

**Affiliations:** Department of Organic Chemistry and Technology, Faculty of Chemical Technology and Biotechnology, 61810 Budapest University of Technology and Economics, Műegyetem rkp. 3, H-1111 Budapest, Hungary

**Keywords:** Process Analytical Technology, Raman Monitoring, Multivariate Analysis, Reaction Feedback Control, Diazotization, Inline Process Monitoring

## Abstract

Inline Raman spectroscopy-based monitoring enables the development of control systems that allow the implementation of Process Analytical Technology (PAT) principles even in chemical reactions, thereby improving quality, efficiency, and safety. There are only a few articles in the literature that discuss such control systems. This may be due to the numerous challenges involved, including the need for robust measurement setups and the development of chemometric algorithms capable of delivering accurate and stable real-time evaluations. Thus, this study presents the Raman spectroscopic monitoring and chemometric-based feedback control of a highly exothermic and potentially hazardous diazotization reaction, namely, the synthesis of phenyldiazonium chloride from aniline, hydrochloric acid, and sodium nitrite. Raman spectra were analyzed offline using Multivariate Curve Resolution-Alternating Least Squares (MCR-ALS) and in real-time using Classical Least Squares (CLS) chemometric methods to monitor and control key reaction components. The developed feedback system successfully controlled concentration levels during both acid–base and diazotization phases. The reduction of the Raman signal-to-noise ratio, induced by the decomposition of the diazonium salt, was also observed. This factor significantly affected the monitoring of the diazotization experiment. Furthermore, a calibration was established to quantify the molar concentrations of key components based on Raman spectra. The calibration experiment successfully demonstrated well-fitted linear molar–spectral relationships for both aniline (*R* ^2^_homogeneous_ = 0.997; *R* ^2^_heterogeneous_ = 0.9947) and aniline hydrochloride (*R* ^2^ = 0.9987). For aniline, the molar–spectral relationship exhibited a distinct change in tendency that precisely correlated with its solubility limit in water, marking the transition from homogeneous solution to emulsion.

## Graphical abstract

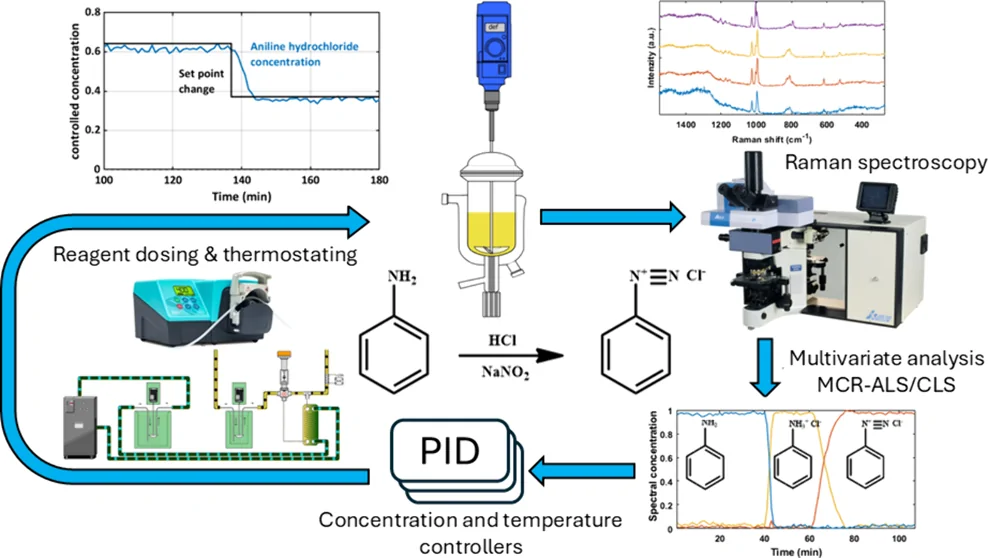

## Introduction

PAT (process analytical technology) has been defined by the U.S. Food and Drug Administration (FDA) as a mechanism for designing, monitoring, analyzing, and controlling pharmaceutical manufacturing processes.^1–3^ PAT solutions are increasingly being adopted not only in the pharmaceutical sector^4^ but also in petrochemical^5^ and food industries,^6^ contributing to optimal and safe operation. Traditional analytical methods, including offline and atline analyses, are used to collect quantitative information. When sampling is required, analyses are conducted at the location of the process (atline), which may lead to longer or shorter delays in data evaluation and processing. In contrast, real-time online or inline measurements included in PAT enable earlier intervention in the process when necessary.^3^ This approach has already been applied to the control of crystallization,^7–10^ granulation,^11–13^ drying,^14–16^ the enzymatic hydrolysis of lactose,^17^ and the oximation reaction.^18^ Feedback control of chemical processes and reactions can have particular importance for highly exothermic and thus potentially dangerous reaction types.^19^

Raman, Fourier transform infrared (FTIR),^20,21^ and near-infrared (NIR)^6,22^ spectroscopy are widely used for process monitoring. Raman spectroscopy is an effective nondestructive method for in situ real-time monitoring of processes, as it can provide a unique “fingerprint” of the reactants involved. Its capability to analyze reaction mixtures containing liquid or even solid phase (e.g., heterogeneous catalysts) Raman-active substances is a significant advantage. It can be used for end point detection or to measure unstable intermediates in the examined mixture.^18^ Raman spectroscopy-based monitoring is mentioned and used in several studies in the literature.^7,17,18,23–28^ Among others, it has already been applied to water quality monitoring by tracking dissolved chemical and microbial contaminants,^24^ monitoring glucose concentrations during ethanol fermentation,^25^ and in pharmaceutical procedures.^29^

Chemometric methods can be used to evaluate the spectrum series generated by the aforementioned measurement techniques. By integrating these spectra evaluation methods into feedback control systems, it enables continuous monitoring of concentrations and adjustment of process parameters (e.g., temperature, dosing rate). It can contribute to the improvement of safety, efficiency, and product quality. To achieve real-time control, the traceability of components in the process must be investigated. If sampling is not possible (e.g., because of unstable intermediate products or rapid side reactions causing the product to transform during offline measurement), chemometric methods are required to obtain the spectra of the specific components. Different chemometric analyses can be used for this purpose, such as Multivariate Curve Resolution-Alternating Least Squares (MCR-ALS).^30^ This is a useful method for identifying components in the spectrum of a reaction mixture that have overlapping chemical bands. With MCR-ALS evaluation, the pure spectra of the different components in the mixture can be estimated, which can be used for the identification of unknown components.^31,32^ This also allows the study of components that cannot be studied in a pure state, for example, for stability reasons.^30^ MCR-ALS analysis can also provide an estimate of the relative spectral concentration changes based on the spectral changes in the examined spectral series. Relative spectral concentration changes can provide useful information about molar concentration changes, particularly when the spectrum of the component differs sufficiently from those of other components of the reaction mixture. On the basis of the result of the traceability investigation, a decision can be made as to whether the information content of the spectra is sufficient to track the individual components. In the next step, a Classical Least Squares (CLS) model can be built that can be used effectively for real-time analysis. To build a CLS model, it is required to provide reference spectra of the components that can be traced during the reaction. Reference spectra can be obtained from direct measurement, MCR-ALS calculation, or a combination of these.^18^

The diazotization reaction examined in this study is the formation of phenyldiazonium chloride from aniline, sodium nitrite, and hydrochloric acid. Diazotization is used to produce aryl-diazonium salts, which are commonly used in the synthesis of azo dyes and in various other organic reactions. The use of aryl-diazonium salts is limited by their instability, which is often reduced by the choice of an appropriate counterion. Many diazonium salts, including aryl-diazonium chlorides, can decompose violently or even explode when they are isolated.

Diazotization was chosen as a model because it is a good example of a dangerous reaction type due to its exothermicity and the pH-dependent nature of the process. Oversaturation of the solution of phenyldiazonium chloride may cause solids to precipitate. Care must be taken to avoid this during experiments, as it can be explosive and has caused several accidents in laboratories.^33^ However, under the appropriate conditions of the experiment, carrying out the reaction has a low risk of danger, as the product is not separated and cannot cause an explosion in an aqueous solution. The reaction is shown in Scheme 1.

**Scheme 1:**
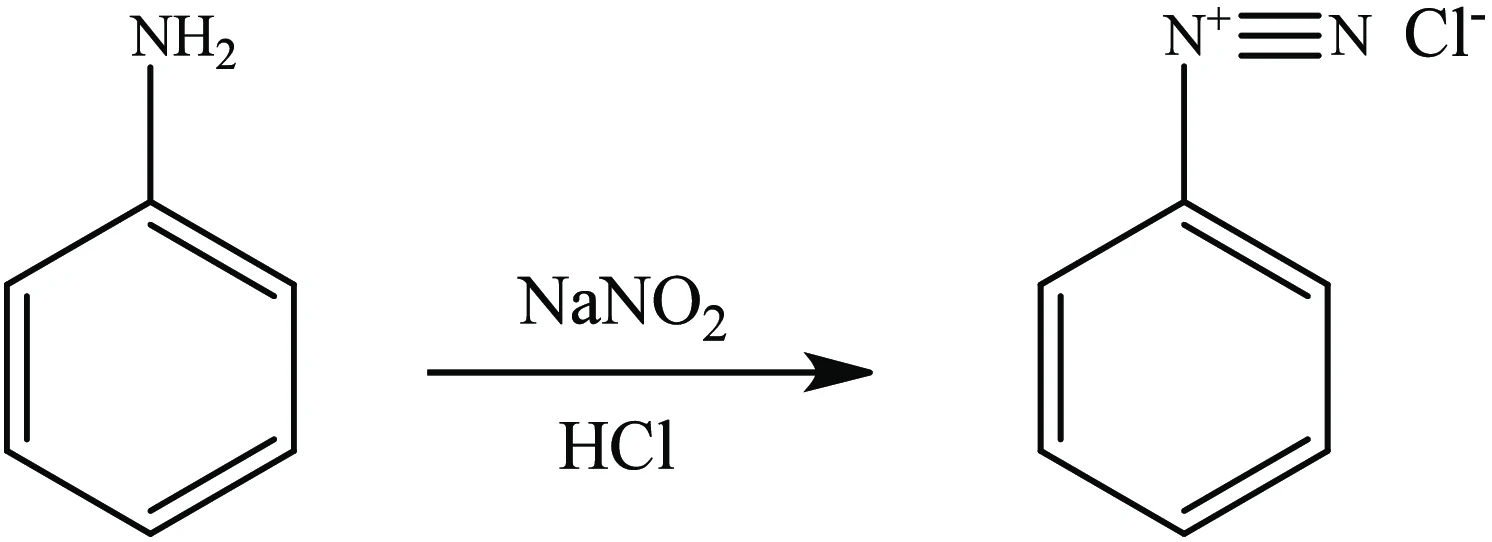
Formation of Phenyldiazonium Chloride from Aniline, Sodium Nitrite, and Hydrochloric Acid

In the experiments, pH 1 was used. Above pH 3, self-coupling may occur as a side reaction, which is the reaction of phenyldiazonium chloride with aniline. The optimal temperature during the reaction is 4 °C. Above this temperature, unfavorable decomposition processes can start. As a result of the decomposition processes among other compounds, chlorobenzene, phenol, 2-nitrophenol, azobenzene, and biphenyl-4-ol can be formed.^34^

In this study, we investigated the feasibility of real-time monitoring of the highly exothermic and potentially hazardous formation of unstable phenyldiazonium chloride from aniline, hydrochloric acid, and sodium nitrite by multivariate analysis of the Raman spectroscopic data. Furthermore, for the first time in the literature, the spectroscopically derived and chemometrically evaluated concentration changes of the reactants and products were integrated into the control loop of this diazotization process. In addition, the correlation between the measured spectral intensities and the molar concentrations was examined to provide a quantitative link between the Raman response and reaction composition.

## Data Analysis Methods

The basic concept of the MCR-ALS method is based on the following eq 1:

1 $$D=CS^{T}+E$$

*D* is a bidirectional data matrix (*m* × *n*) from which two matrices are constructed: concentration *C* (*m* × *k*) and pure component matrix *S* ^T^ (*k* × *n*), where *k* components are searched. *E* is an error matrix containing the residual data. The MCR-ALS method can calculate the concentration of each component given the initial concentrations. Knowing these concentrations (matrix *C*) and the matrix containing the spectral sequences *D*, the spectra of the pure components contained in the *S* ^T^ matrix can be determined. If the reconstructed data matrix (*C* *S* ^T^) does not sufficiently reproduce the measured data matrix *D*, the estimation of *C* and *S* ^T^ is alternated iteratively until convergence is achieved and the residual matrix *E* is minimized.^30^

CLS is a similar method that can model the examined spectrum of a multicomponent mixture as a linear combination (weighted average) of the reference spectra of the individual components in real time. Given the reference spectra, the weighting factors of the aforementioned linear combination can be calculated by simple mathematical operations and, if necessary, after appropriate scaling can be used to estimate concentrations.^30^

While MCR-ALS utilizes the spectral series of the reaction mixture to evaluate spectral changes over time and estimate the pure component spectra, CLS employs known reference spectra to reconstruct the spectra of the reaction mixture and estimate the component concentrations even in real time.

The real-time chemometric evaluation of data measured during monitoring provides information that can contribute to the optimal operation of the process from a quality, performance, and safety point of view. Feedback from these calculated parameters can be used as input to control loops whose development and practical testing are rarely published in the literature.^18,34^

## Experimental Section

### Equipment

The experiments were conducted using a laboratory reactor system (see Figure 1) under computer control, including the following key components. The reactor module in the setup supplied by Normag Labor- and Prozesstechnik GmbH (Ilmenau, Germany) consisted of a 150 mL jacketed glass vessel that has a circular window in its jacket for the Raman probe. The experimental setup used for the concentration-based feedback control is shown in Figure 2.

**Figure 1:**
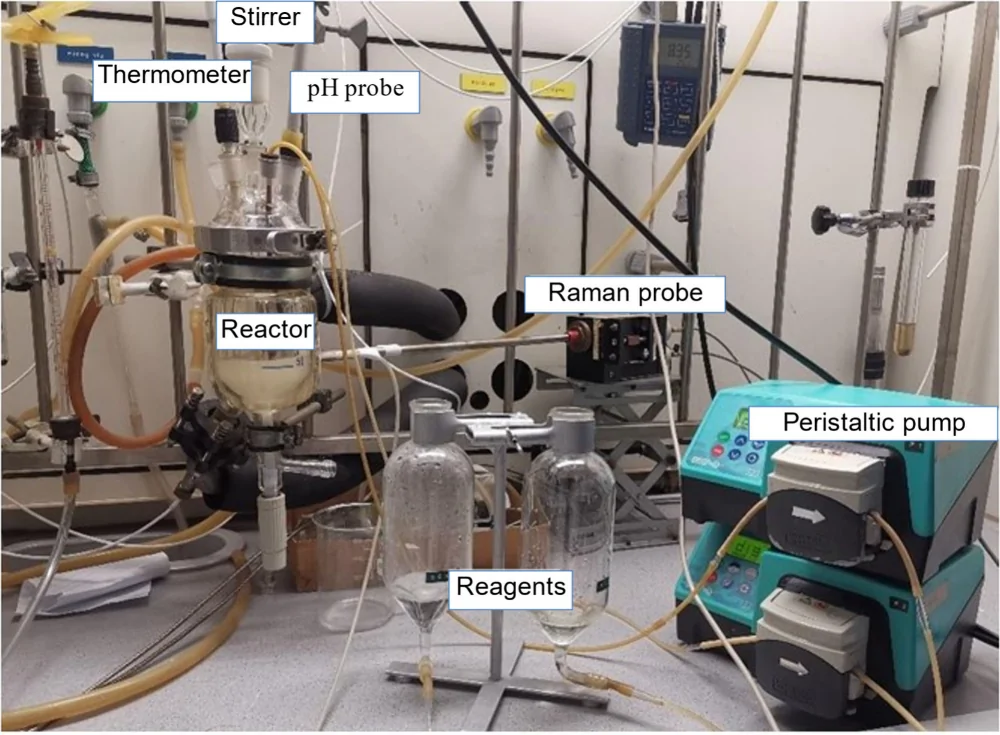
Picture of the experimental setup.

**Figure 2:**
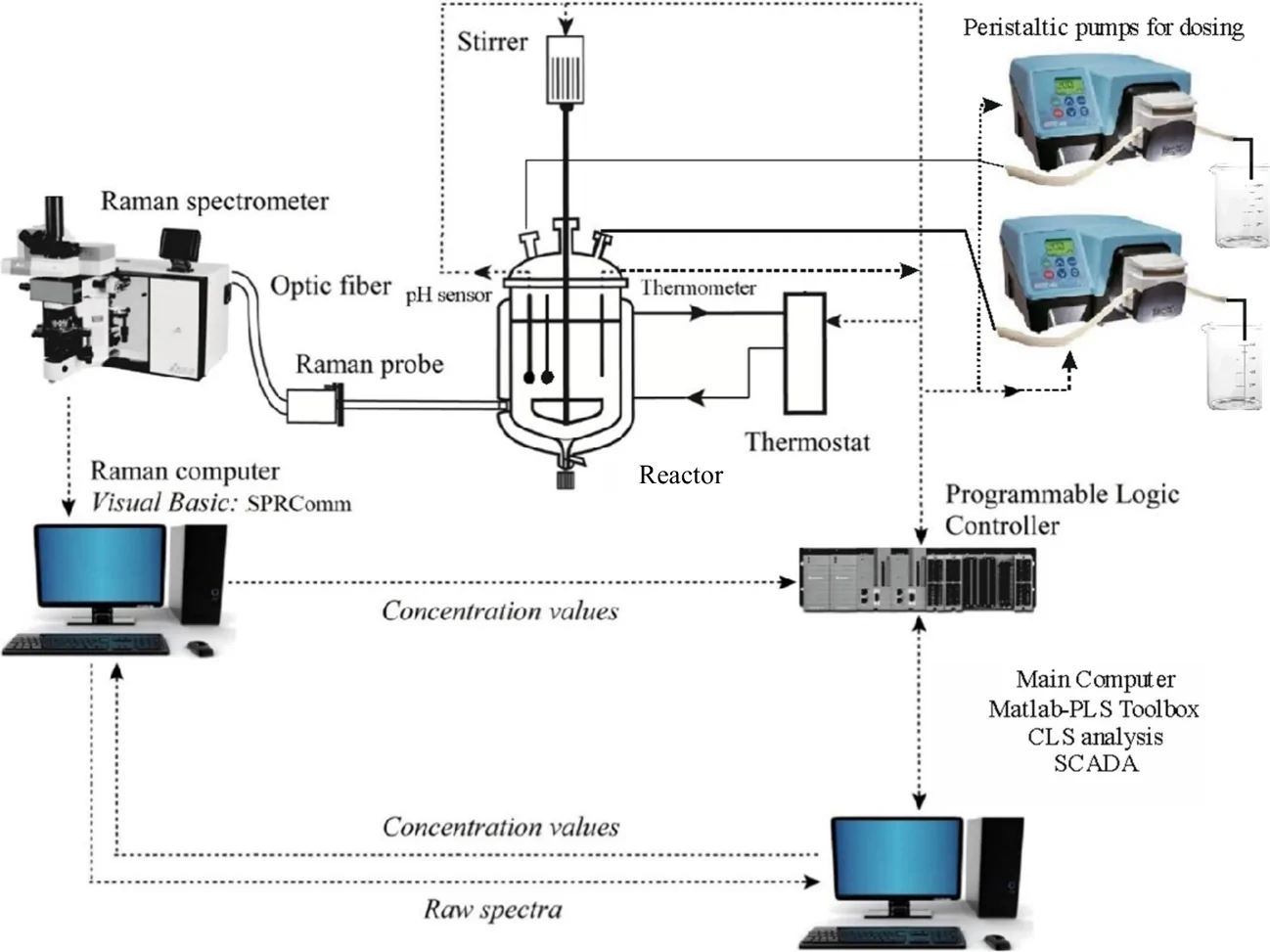
Experimental setup for concentration-based feedback control (SCADA: Supervisory Control and Data Acquisition).

Control operations were managed through a Stardom FCN-type programmable logic controller (PLC) manufactured by Yokogawa Electric Corporation (Tokyo, Japan). Temperature control within the reactor was achieved by a PID controller, which adjusted the temperature of the circulating oil in the reactor’s jacket. The dosing system featured a Watson Marlow 300 series laboratory peristaltic pump. Stirring was achieved using a Eurostar power control-visc type stirrer from IKA-Werke GmbH & Co. KG (Staufen, Germany). The dosing system was also controlled by the PLC. The pH monitoring was conducted using a combined glass electrode and a Portamess 913 pH meter from Knick Elektronische Messgeräte GmbH & Co. KG (Berlin, Germany). Inline noninvasive Raman spectra were measured using a Raman spectrometer (Horiba Jobin Yvon SAS, Kyoto, Japan) coupled with a fiber-optic probe. Real-time Raman spectra were acquired within the spectral range of 270.5–1551.4 cm^–1^ using a Peltier-cooled Charge-Coupled Device (CCD) detector. A 400 mW diode laser (785 nm) served as the light source with an exposure time of 30 s per spectrum and an accumulation number of 2.

### Materials

Aniline (VWR, ≥99%), NaNO_2_ (Sigma-Aldrich, ≥98), and HCl solution (Sigma-Aldrich, 37%) were used during experiments.

### Experiments

In the first phase, an aniline–water heterogeneous mixture (6.5 mL of aniline in 50 mL of H_2_O) was cooled to 4 °C by a control loop and adjusted to the optimum pH value, which is strongly acidic (pH = 1), by adding hydrochloric acid solution (22% solution made by diluting 37%). During the addition, the heterogeneous mixture gradually became homogeneous, while aniline hydrochloride was formed. The second phase started when the control algorithm started dosing the NaNO_2_ reagent solution, while intensive cooling was provided through the reactor jacket (Stirring rate = 310 rpm). While the reaction was carried out, Raman spectra were recorded every minute. After the experiment was completed, the spectra were evaluated. The spectra were preprocessed using Matlab (R2024b, The MathWorks Inc., USA) and the PLS-Toolbox (Eigenvector Research, Inc., Manson, WA, USA) software package by the following steps:

1. Smoothing (Savitzky–Golay filter) with parameters: filter width = 15 and polynomial order = 2.
2. Baseline correction (Automatic Whittaker Filter) with parameters: λ = 100,000 and *P* = 0.001.
3. Normalization was used with the length of the curve = 1.

In the calibration experiment, 75 mL of distilled water was cooled to 4 °C in the reactor before the start of spectral acquisition. 10.74 g of aniline was added in multiple steps. After the completion of aniline addition, 22 wt % hydrochloric acid solution was also added stepwise until the pH reached 1, as is required for the diazotization reaction. 8.00 g of sodium nitrite in 15 wt % solution was then added in multiple steps. During the diazotization, the temperature was maintained within the required diazotization range by cooling the jacket, and the pH was adjusted by dosing a 22 wt % hydrochloric acid solution.

## Results and Discussion

The primary objectives of this study were to assess the real-time monitoring of the diazotization reaction leading to the formation of phenyldiazonium chloride and then develop a spectroscopy-based control system using Raman spectroscopy and multivariate data analysis. Before implementation of the feedback control system, the traceability of the individual components had to be investigated through offline MCR-ALS evaluation.

### Monitoring of the Diazotization Reaction

Raman spectra were recorded every minute during the diazotization experiment. The time-resolved Raman spectra of the reaction are presented in Figure 3 as three-dimensional plots. The spectral number also served as a time stamp as it indicated the elapsed time in minutes from the start of the experiment. The hydrochloric acid solution was added at the 40th minute, while the sodium nitrite solution was added at the 60th minute. Spectral changes were observed after each addition.

**Figure 3:**
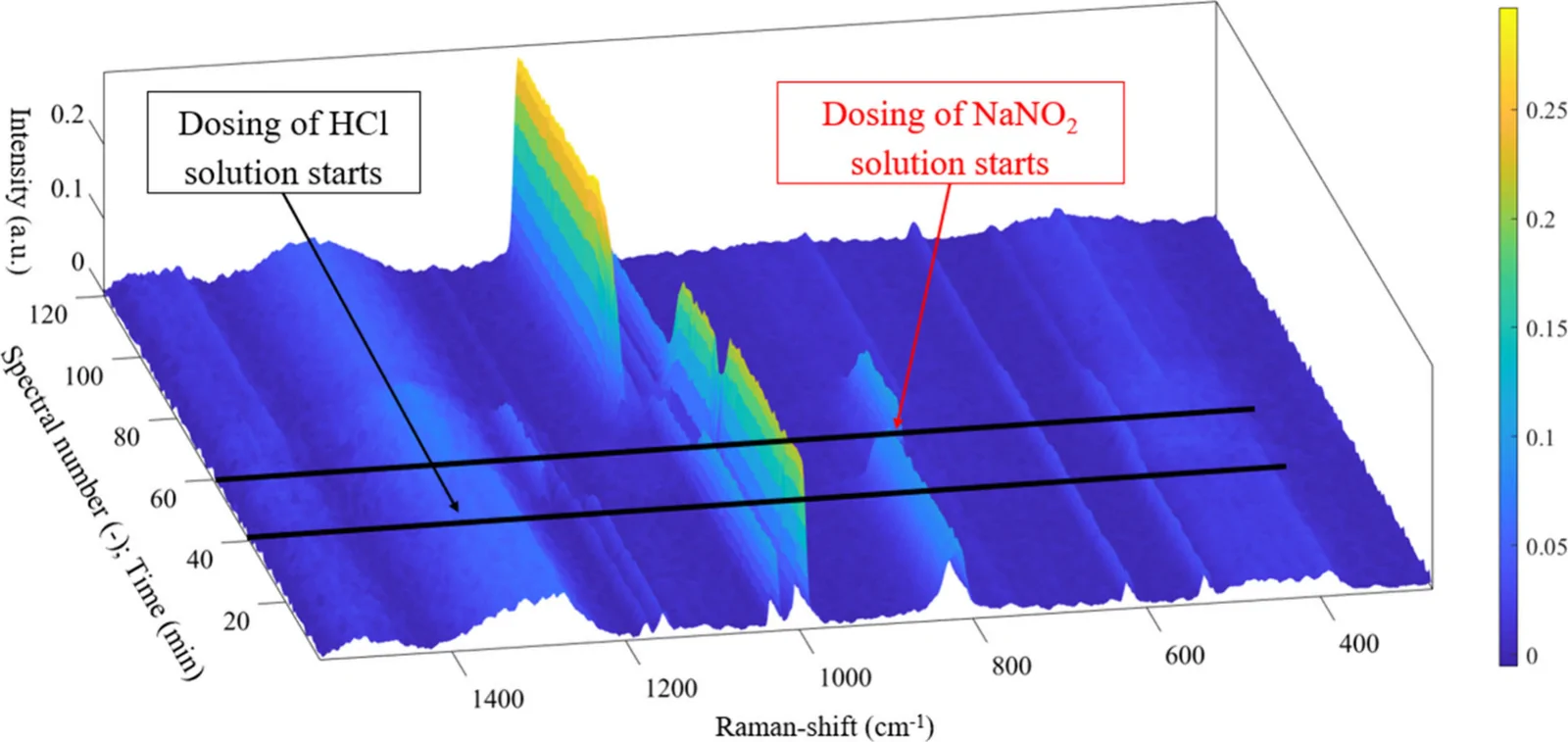
3D visualization of the diazotization experiment.

Using an MCR-ALS chemometric evaluation, the possible reference spectra of the main components were identified (Figure 4). Comparison of the calculated pure-component spectra with measured solution spectra allowed the identification of three components in the reaction mixture: aniline, aniline hydrochloride, and phenyldiazonium chloride (Figure 4).

**Figure 4:**
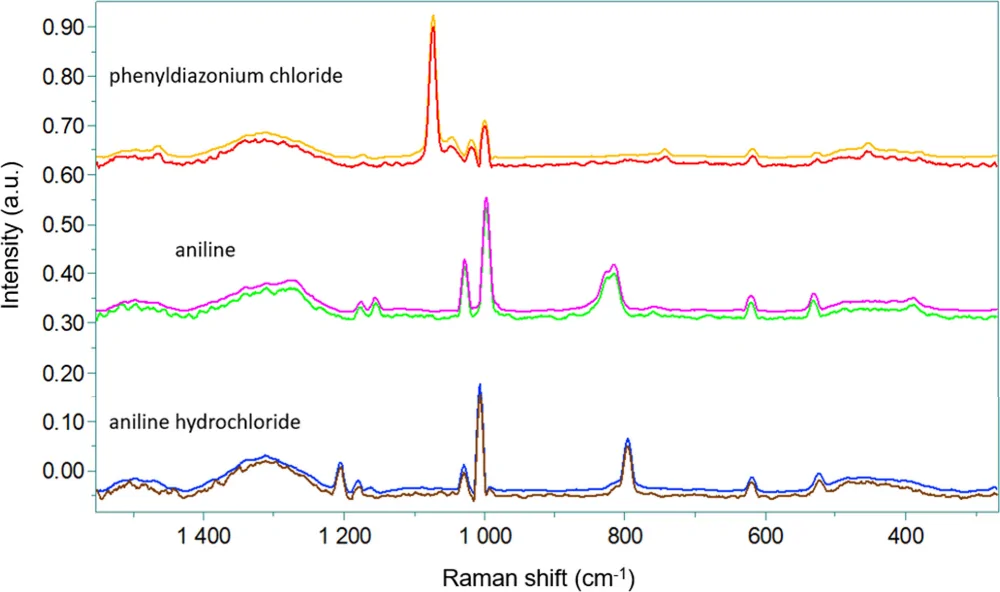
Comparison of Raman spectra estimated by the MCR-ALS method (upper) and measured spectra in an aqueous solution (lower).

The spectral concentration vs spectral number diagram, shown in Figure 5, was calculated using the MCR-ALS analysis method by the PLS toolbox software package detailed in the Experimental Section. Figure 5 shows that during the aniline hydrochloride formation due to the addition of hydrochloric acid to aniline–water a heterogeneous system can be identified. This was considered the first phase of the technology (0–60 min). In the second phase (60–110 min), the formation of phenyldiazonium chloride due to the addition of NaNO_2_ solution is also successfully detected.

**Figure 5:**
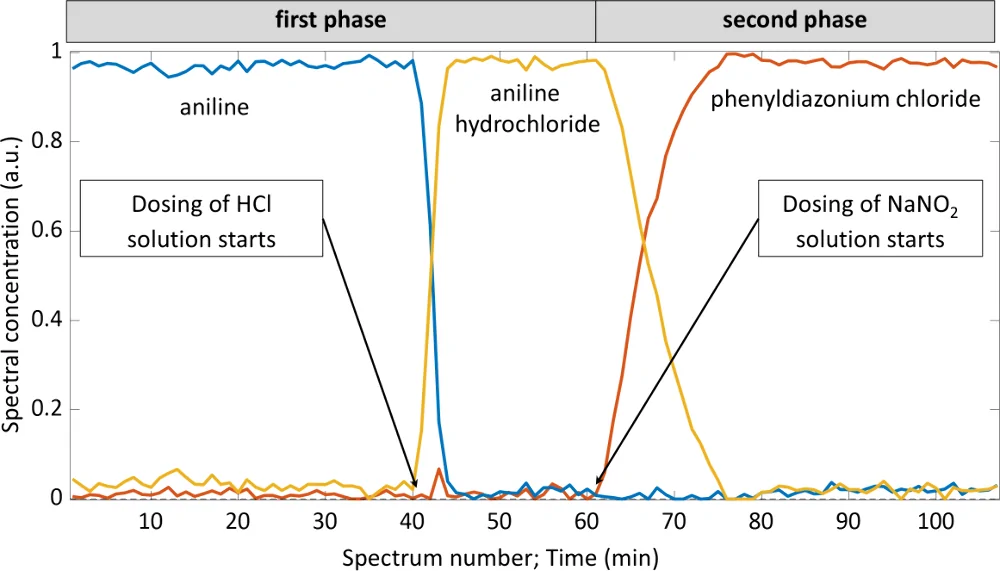
Spectral concentration–time diagram of the monitoring experiment based on the offline MCR-ALS analysis of Raman spectra.

### Feedback Control of the Diazotization Reaction

The CLS model used for real-time reaction control required reference spectra of the traceable components. Three components were included in the analysis: Representative spectra for each of the components could be selected from the spectral series in which only one component was present apart from water. At the beginning of the first phase of the experiment, one selected spectrum was characteristic of aniline during the monitoring of the water–aniline heterogeneous system. At the end of the first phase of the experiment, a spectrum representing aniline hydrochloride was isolated. Finally, only Raman peaks belonging to phenyldiazonium chloride were found at the end of the second dosing phase. The reference spectra required for the CLS model were selected from these sections of the experiment.

To demonstrate the effectiveness of the real-time spectroscopy-based feedback concentration control, a unique experimental setting was developed and applied to this model reaction. In the first and second phases, concentration levels were set, and the control system had to adjust them by adding the necessary dose of the reagent. After setting each concentration set point, the control system stabilized the temperature and pH and subsequently performed the next concentration-level adjustment. A schematic of the equipment used for the control system is shown in the Experimental Section.

In the first phase of the experiment, 2 such concentration set point values were set using the C0 controller. Subsequently, the control system switched off the C0 concentration controller and switched on the C2 pH controller, which set the pH to an optimum value for the second phase. This control step also increased the concentration of aniline hydrochloride, causing a further step in the spectral concentration. In the second phase, 3 concentration set point values were set. The system was configured to operate four simultaneous control loops during the experiment, as can be seen in Figure 6.

**Figure 6:**
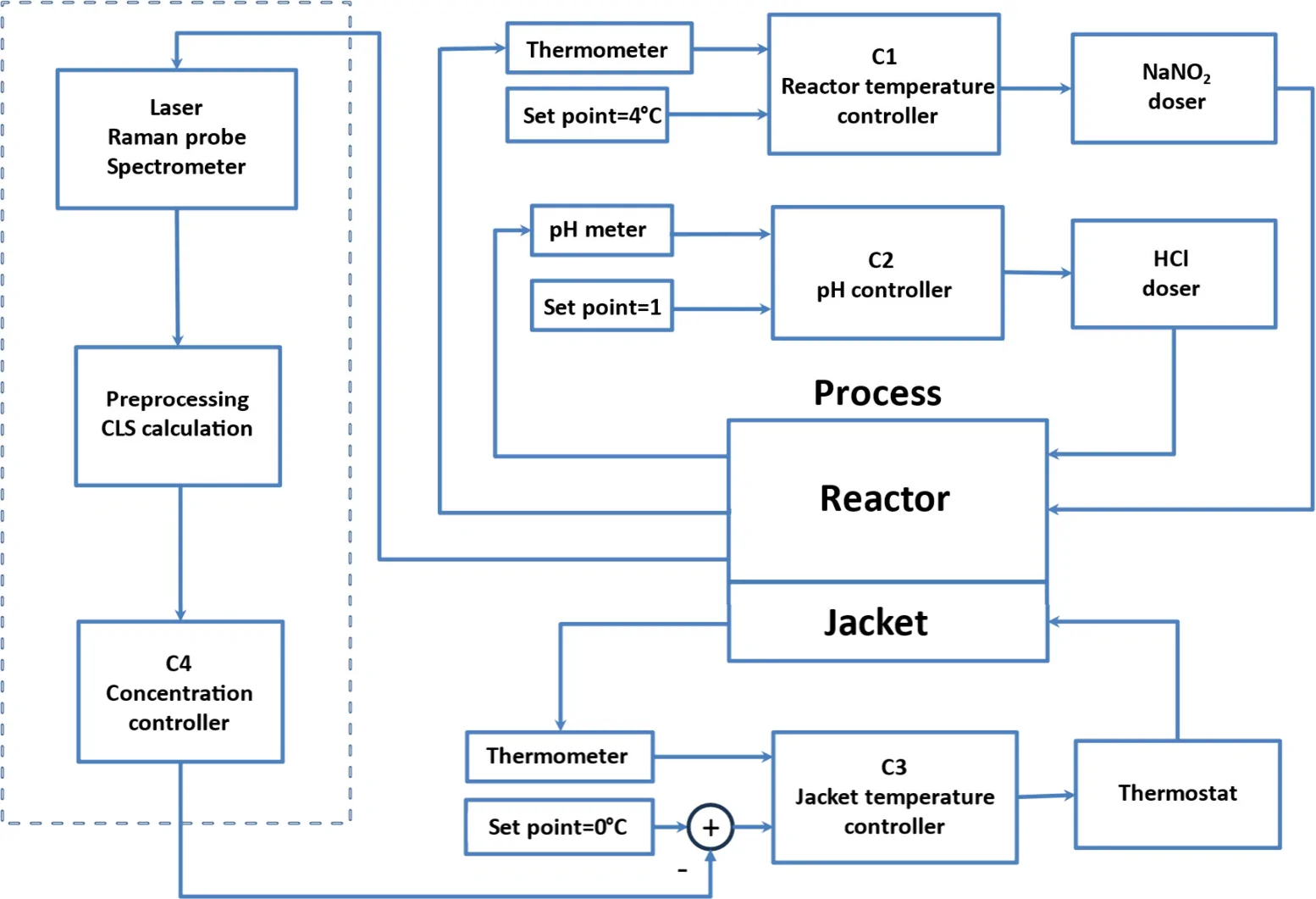
Schematic drawing of the control loops in the spectroscopy-based feedback control experiment for the reaction.

The C1 reactor temperature controller was employed to control the addition of the NaNO_2_ solution during the second phase of the experiment. As the exothermic diazotization reaction begins, heat is released. This control loop compensates for the temperature drop caused by the cooling jacket by changing the reagent dosing rate with a NaNO_2_ dosing pump.

The C2 pH controller was used to control the pH level. To maintain a pH of 1 in the reaction mixture, the regulator controlled the rate of the HCl dosing pump feeding the hydrochloric acid solution.

The C3 jacket temperature controller and the C4 concentration controller functioned as cascades.

The cascade was used to prevent possible freezing and crystallization processes caused by overcooling of the reaction mixture. The C3 jacket temperature controller ensured that the temperature of the circulating silicone oil was suitable for heating or cooling the reactor.

The C4 control loop controls the concentration of a selected component in the reaction mixture. In our case, the selected components were aniline in the first phase and phenyldiazonium chloride in the second phase.

Using the concentration value calculated by the chemometric method and the predetermined set point, the controller calculated the control output (temperature shift) required to modify the C3 set point.

Until the set point is reached, the C4 controller reduces the jacket temperature via the C3 controller. This triggers the dosing of NaNO_2_ and HCl via C1 and C2. However, when the desired concentration is reached, the output of C4 becomes zero, and therefore, the C3 controller only maintains the temperature in the jacket that is necessary to keep the reactor at 4 °C.

In our experimental setup, we had to set the temperature in the jacket to 0 °C so that the temperature of the reaction mixture would remain at 4 °C over a longer period of time.

A PLC program was prepared to implement the phases of the technology, which consists of salt formation and diazotization. The sequence of instructions performed in the experiment is shown in Figure 7.

**Figure 7:**
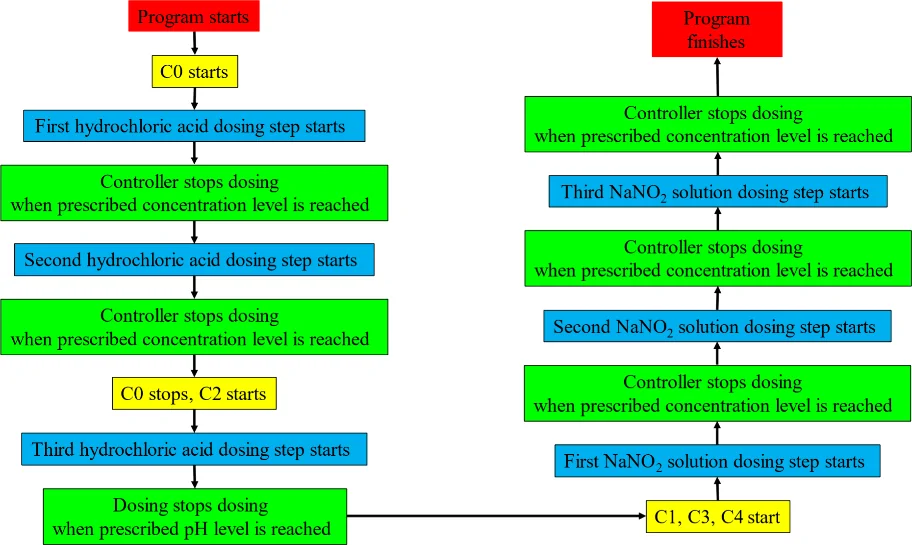
Schematic structure of the control algorithm sequence.

The experiment was carried out by the control algorithm shown in Figure 7. In the first phase, the algorithm successfully performed the spectroscopy-based concentration feedback control. The aniline concentration level was adjusted in two steps to the specified set points of 0.7 and 0.25, and then, the pH level was adjusted to 1. During the experiment, we observed a significant overshoot in the concentration steps. This was due to the controller not being optimally tuned.

Figure 8 shows that the spectral concentration data follow the expected trend: by the end of the first phase, neither the starting material nor the diazonium salt is present in the reactor, while the concentration of the intermediate, aniline hydrochloride, reaches its maximum. The spectral concentration values observed at the beginning and end of the phase indicated that the CLS method functioned properly.

**Figure 8:**
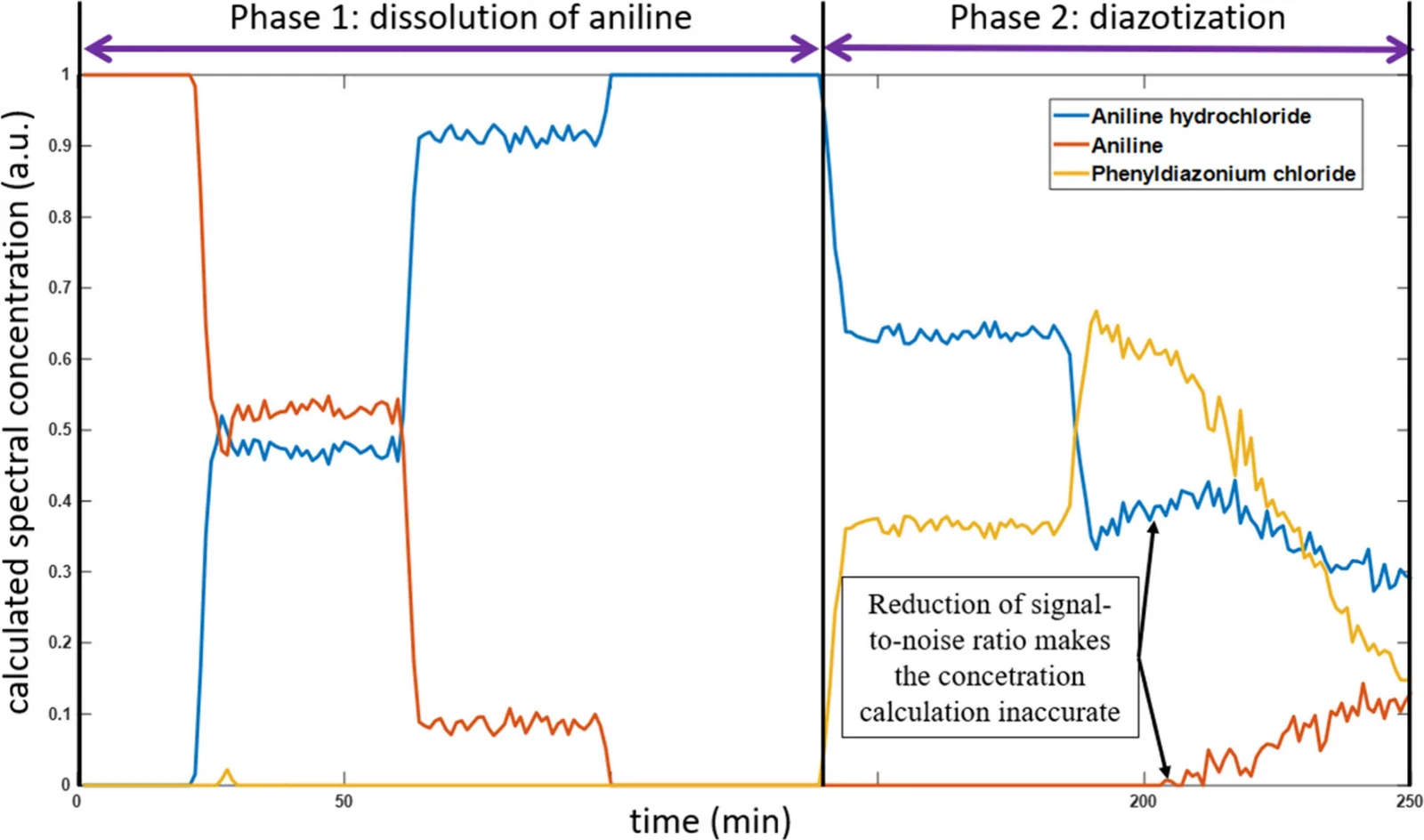
CLS concentration profiles of the Raman spectroscopy-based concentration control reaction.

Spectroscopy-based concentration feedback control was also achieved in the second phase, as shown in Figure 8.

During the second phase, a baseline increase and a declining signal-to-noise ratio were observed in the spectra, presumably due to the fluorescent property of the decomposition products of the diazonium salt, which made it unfeasible for the applied model to continue monitoring the concentration profiles.

### Calibration Experiment of the Diazotization Reaction

The spectral concentration profiles determined in the experiments are based on the chemometric analysis of spectra. However, spectral concentration is not so widely used compared with molar concentration. Therefore, to understand the possible relationship between the spectral and molar concentrations, we performed a calibration experiment.

In the calibration experiment, known amounts of aniline were added in multiple steps to the reactor, which initially contained only distilled water. Subsequently, hydrochloric acid was introduced in multiple steps as well, followed by the stepwise addition of a known amount of a sodium nitrite solution. During the calibration experiment, we assumed that the reactions take place quantitatively and no byproducts are formed. With these assumptions, it was possible to avoid the use of diazonium salts in their explosive pure form.

The spectral concentration values for each dosing step were determined by the CLS calculation. Spectral concentration values can be calculated for the aniline component during the aniline dosing (ascending period) and also during the hydrochloric acid dosing (descending period) in the aniline dissolution phase. A calibration curve can be made for both the ascending and descending periods. The molar concentrations were determined from the known amounts of reactants during the dosage steps.

The molar concentration values calculated from the dosages in each step were plotted as a function of the spectral concentration values calculated by the CLS model, as shown in Figure 9.

**Figure 9:**
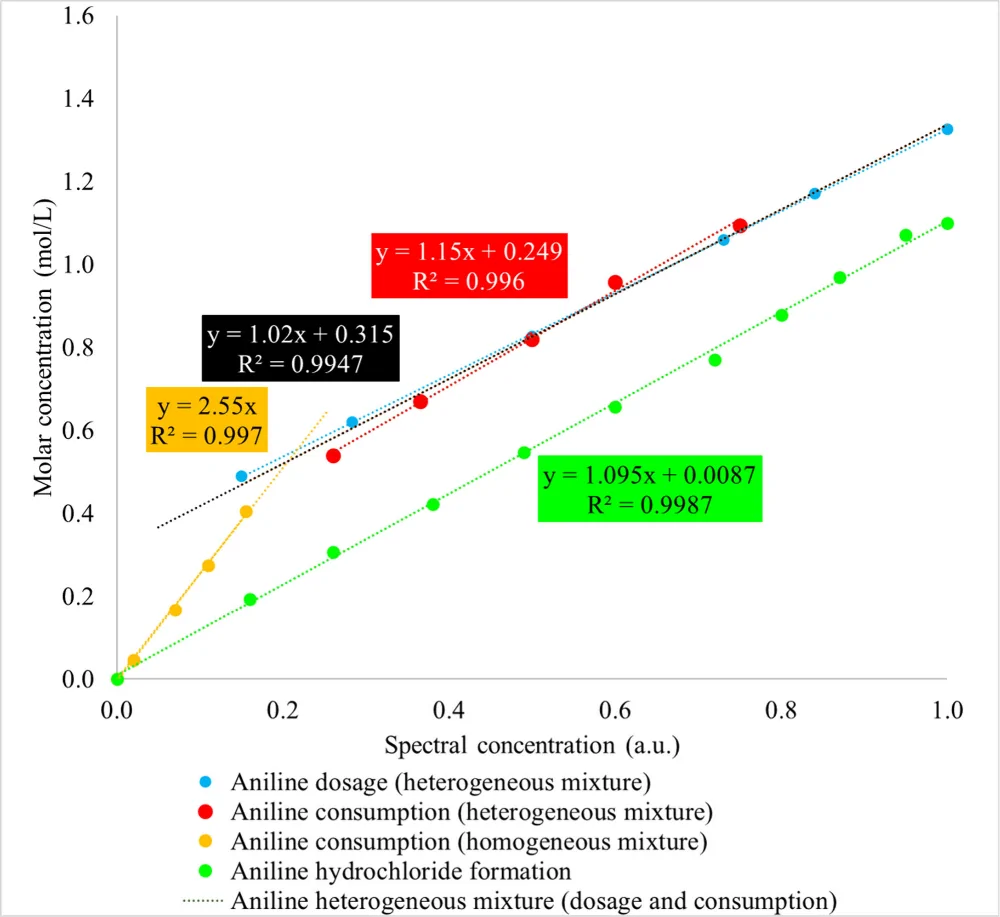
Calibration of aniline and aniline hydrochloride concentrations by Raman spectroscopy.

During the CLS analysis, we used the spectra of the aniline–water heterogeneous mixture, aniline hydrochloride solution, and phenyldiazonium chloride solution as references. In addition to these, a fourth reference spectrum was introduced. At the beginning of the experiment, when none of the above components had yet been added to the mixture, only water was present, which has no Raman active bands. Consequently, the CLS method performs poorly because it tries to reconstruct a baseline without characteristic bands from characteristic spectra. In this case, the spectral concentrations of the components appear to be random noise. To avoid this, we also entered a homogeneous aniline–water mixture with a low aniline concentration (1%) into the CLS model as a reference spectrum.

The points of aniline addition and consumption did not fall on a straight line in the calibration diagram. Three sections were distinguished. One section was associated with the addition and two sections were for the consumption of aniline. A breakpoint was observed at 0.4 mol/L following the first four (amber colored) data points in the lower concentration range. At concentrations below the breakpoint, the reaction mixture was in homogeneous form. At concentrations above the breakpoint, the reaction mixture was in heterogeneous form. During the consumption of aniline, the heterogeneous mixture also contains dissolved salt, so the calibration line deviates slightly from the addition section. Based on these observations, treating homogeneous and heterogeneous regions separately, the calibration lines showed good fit (*R* ^2^ = 0.997; 0.9999; 0.996).

The relationship between molar and spectral concentrations can be approximated as linear within the concentration ranges of 0 to 0.5 mol/L and 0.5 to 1.33 mol/L. Notably, the intersection of the two linear fits corresponds to the solubility limit of aniline in water (3.6 g/100 mL) with the mixture appearing homogeneous below and heterogeneous (emulsion) above this point.

The calibration points for the aniline hydrochloride intermediate are also listed in Figure 9. It can be seen that the relationship between the molar and spectral concentrations of aniline hydrochloride is directly proportional during the hydrochloric acid addition phase (*R* ^2^ = 0.999).

During sodium nitrite addition, a worsening signal-to-noise ratio and rising baseline were observed. Therefore, many dosing steps during sodium nitrite addition could not be monitored by Raman spectroscopy. For this reason, no calibration curve was obtained for the diazonium salt.

### Evaluation of the CLS Model Fitting for the Diazotization Experiment

Matlab’s correlation function “corr”, which calculates the Pearson’s correlation coefficient, can be used to quantitatively characterize the goodness of fit of the CLS model. The correlation of the model can be calculated by comparing the linear combination of the reference spectra with the spectra of the measured reaction mixture.

As shown in Figure 10, the correlation value calculated during the experiment is close to 1, confirming the good fit of the model up to 200 min. Thereafter, the correlation value decreased in accordance with the decreasing signal-to-noise ratio. The figure shows that the progressive decline of the signal-to-noise ratio became so severe over time that the characteristic spectral features eventually disappeared completely. With such an extensive loss of spectral information, CLS-based chemometric evaluation was not able to provide reliable results. Although more adaptive and carefully optimized spectral preprocessing strategies could presumably extend the period over which the process remains trackable, the extent of spectral information loss shown in the figure suggests that monitoring capability would inevitably be lost at a later stage.

**Figure 10:**
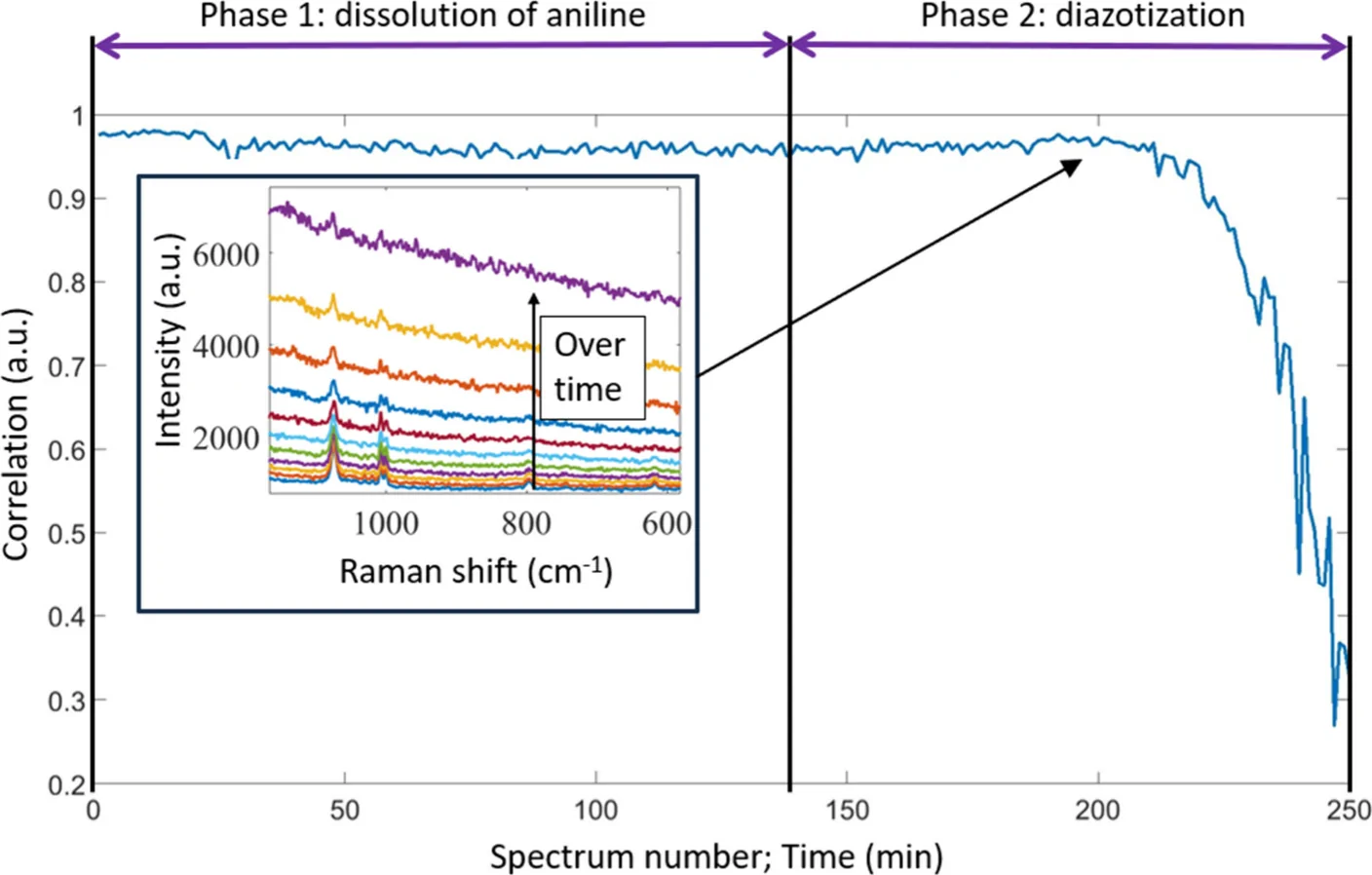
Correlation of the model used in real-time spectroscopy-based concentration control.

Comparing the current results with similar studies in the literature, it can be said that spectroscopic methods are widely used to monitor pharmaceutical technology processes (drying, homogenization, and crystallization). There are significantly fewer examples of chemical processes being monitored in real-time using an inline method. In 2002, a method was developed for real-time monitoring of the esterification of acetic acid and methanol using NIR spectroscopy with PLS and PCA methods,^35^ and in 2024, in-line monitoring of the synthesis of the active ingredient intermediate lifitegrast was implemented using NIR and Raman spectroscopy and the PLS method.^36^ In 2013, a method was developed using PCA to monitor the transesterification process in biodiesel synthesis.^37^ Our research group has implemented a true Raman-spectroscopy-based feedback control system. One example of this was the acetone oximation process in which the concentration of the unstable intermediate was maintained at a constant level using a control system that prevented its unwanted accumulation.^18^ Another example from our research group involves a complex, nonlinear bioprocess in which the lactose concentration was maintained at a constant level of 20 g/L.^17^ In terms of its methodology and objectives (safe control of an exothermic process based on Raman spectroscopy), the diazotization reaction most closely resembles the oximation reaction. However, the reaction is more complex and requires simultaneous control of both temperature and pH. In this study, it was revealed that Raman-based monitoring in this system has a practical limitation: at the end of the process, the rise in the spectral baseline and the decline in the signal-to-noise ratio reduced the reliability of the monitoring. This drastic signal degradation has been less critical in other reactions that have been studied in the literature.

Altogether, this study demonstrates the successful implementation of real-time Raman spectroscopic monitoring and chemometric-based feedback control for a hazardous diazotization reaction, showcasing the potential of PAT tools to enhance process safety and efficiency. This is supported by a calibration experiment that establishes the quantitative relationship between the spectral and molar concentrations of key components.

## Conclusion

The principles of process analytical technologies, as defined by the FDA, are increasingly being adopted across a wide range of industries thanks to their benefits regarding the monitoring and therefore the possible control of processes. The objective of this paper was to develop spectroscopy-based monitoring of a potentially hazardous diazotization reaction. Knowing the component concentrations in the reaction mixture supports process control.

During the experiments, spectra were recorded by Raman spectroscopy and evaluated by MCR-ALS (offline) and CLS (inline) chemometrics. It was found that Raman spectroscopy is suitable for monitoring the process, and it is possible to identify the three main components of the reaction mixture: aniline, aniline hydrochloride, and phenyldiazonium chloride. A rising baseline in the Raman spectra was observed during the dosing of the NaNO_2_ solution, which could be attributed to the fluorescent nature of the decomposition products of the diazonium salt. To determine concentrations in real time, the CLS method was used. For real-time control, a process control algorithm was developed that enabled the control of aniline hydrochloride and phenyldiazonium chloride formation in a PAT system based on preprogrammed concentration values. The experiment implementing concentration-based control consisted of two parts: an initial acid–base reaction followed by a diazotization reaction. In these phases, the control system adjusted the predefined concentration levels. During the first phase, the spectroscopy-based control system successfully reached the prescribed concentration level two times. Similarly, in the subsequent second phase, the system achieved a target concentration level twice. However, following these adjustments, unrealistic concentration data were observed because of the declining signal-to-noise ratio in the Raman spectra. The results presented in this study successfully demonstrated that diazotization processes can be effectively monitored and controlled using Raman spectroscopy until the amount of byproducts increases.

The limit of Raman-based traceability is sensitively indicated by a decrease in the correlation coefficient.

Furthermore, the methodology presented here integrates the spectroscopic concentration data into a control loop. This approach can provide a useful basis for further studies on PAT-oriented spectroscopic control of other complex or hazardous pharmaceutical and fine chemical processes.

More broadly, this work illustrates how PAT-oriented spectroscopic tools may contribute to QbD-driven process development by expanding the possibilities for real-time monitoring and control.
